# Supplementary material for: Designed optogenetic tool for bridging single-neuronal multimodal information in intact animals
Source: Nat Commun. 2025 Aug 20;16:7764. doi: 10.1038/s41467-025-62938-w (PMC12368189; doi:10.1038/s41467-025-62938-w)
Supplement: Supplementary file 1 — Supplementary Information [file 41467_2025_62938_MOESM1_ESM.pdf]

Designed optogenetic tool for bridging single-neuronal multimodal information in intact animals

Rong-Kun Tao[1,2,*,#], Le Sun[1,3,*], Yu Qian[1,3], Yi-Ming Huang[4], Yu-Han Chen[1,5], Chun-Yu Guan[6], Ming-Cang Wang[6], Yi-Di Sun[1] & Jiu-Lin Du[1,3,4,#]

[1] Institute of Neuroscience, Laboratory of Brain Cognition and Brain-Inspired Intelligence Technology, Center for Excellence in Brain Science and Intelligence Technology, Chinese Academy of Sciences, 320 Yue-Yang Road, Shanghai 200031, China

[2] Clinical Research Institute, The First Affiliated Hospital of Xiamen University, School of Medicine, Xiamen University, 4221 South Xiang-An Road, Fujian 361102, China

[3] University of Chinese Academy of Sciences, 19A Yu-Quan Road, Beijing 100049, China

[4] School of Life Science and Technology, ShanghaiTech University, 319 Yue-Yang Road, Shanghai 200031, China

[5] School of Life Science and Technology, ShanghaiTech University, 393 Middle Hua-Xia Road, Shanghai 201210, China

[6] Department of Anesthesiology, Taizhou Hospital of Zhejiang Province Affiliated to Wenzhou Medical University, 150 Xi-Men Road, Zhejiang 317000, China

[*] These authors contributed equally.

[#] Correspondence E-mail: taorongkun@xmu.edu.cn; forestdu@ion.ac.cn

Supplementary Figures

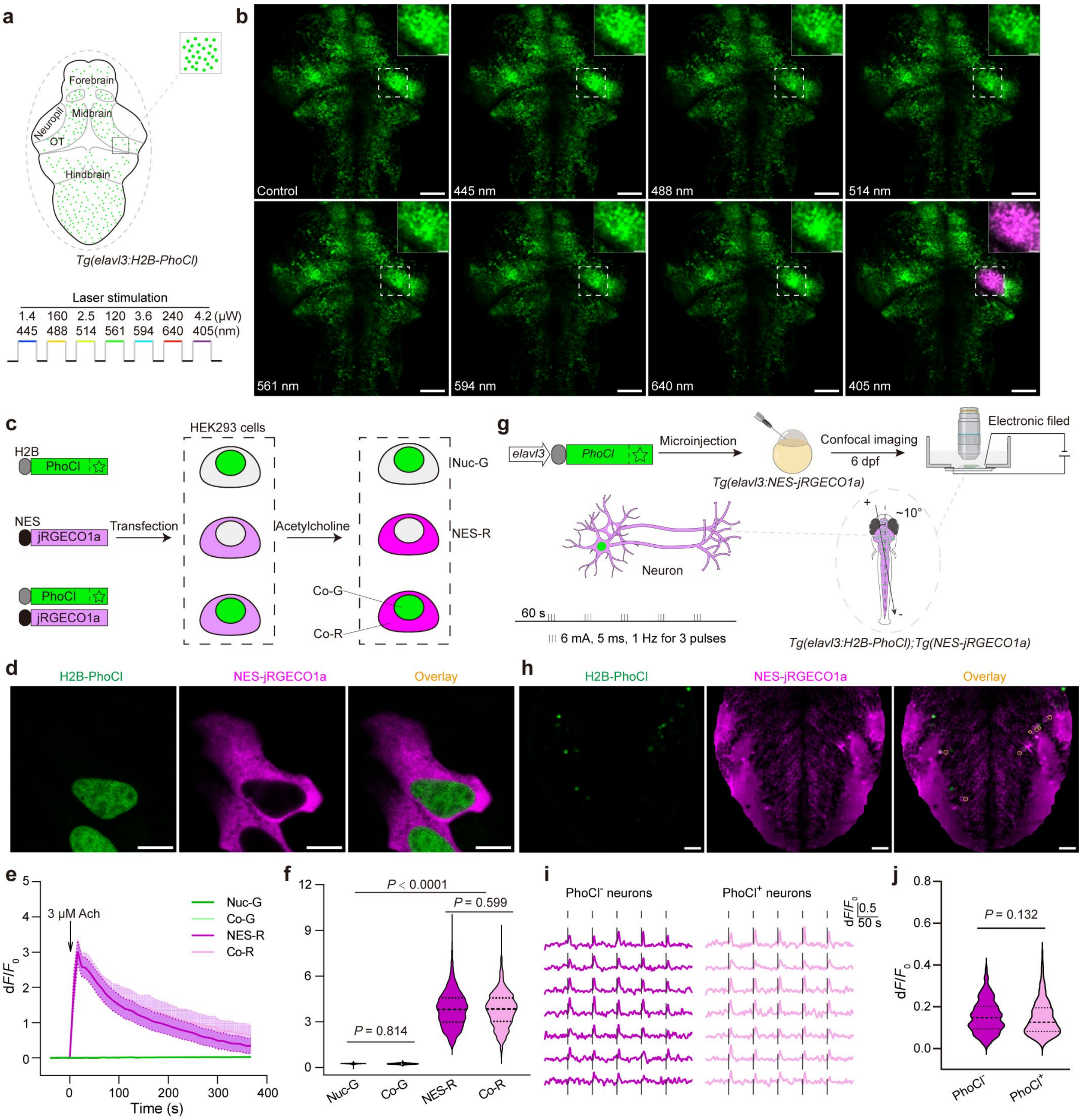

Supplementary Fig. 1. Photo-conversion properties of H2B-PhoCl and its effects on intracellular calcium activities in cell cultures and *in vivo*.

a, Schematic illustrating the photo-conversion properties of H2B-PhoCl in a selected region. Sequential laser pulses (405nm: 4.2μW, 445nm: 1.4μW, 488nm: 160μW, 514nm: 2.5μW, 561nm: 120μW, 594nm: 3.6μW, 640nm: 240μW) applied for 30s.

b, Fluorescence images of larval zebrafish expressing H2B-PhoCl after illumination with different laser wavelengths (n = 6 fish). Zoomed-in views of the selected region (dashed square) are shown. Only 405-nm laser activates PhoCl. Scale bars: 50 µm (full); 10 µm (zoomed).

c, Schematic of the experimental design showing the fluorescence response of HEK293 cells expressing H2B-PhoCl (Nuc-G), NES-jRGECO1a (NES-R), both H2B-PhoCl (Co-G) and NES-jRGECO1a (Co-R), stimulated with 3 µM acetylcholine (Ach).

d-f, Representative images (d), fluorescence traces (e), and grouped analysis of average responses (f) in HEK293 cells expressing nuclear PhoCl and/or NES-jRGECO1a after Ach stimulation (n = 4 experiments). Scale bars: 10 µm. No significant effect of H2B-PhoCl on calcium response was observed. The dashed line represents the median, and the lower and upper dashed lines indicate the 1st and 3rd quartile. Unpaired one-tailed Student's *t*-test for Nuc-G vs. Co-G; NES-R vs. Co-R, and two-way ANOVA for four groups.

g, Schematic of the experiment testing the effect of H2B-PhoCl on neural activity in 6-dpf larval zebrafish expressing *Tg(elavl3:NES-jRGECO1a)*. Neurons were stimulated using an electronic field (6 mA, 5 ms, 1 Hz, 3 trials, 60 s interval).

h, Representative images of neurons expressing H2B-PhoCl in the *Tg(elavl3:NES-jRGECO1a)* fishline (n = 12 fish). The calcium response in PhoCl^{+} neurons (pink) was compared to adjacent PhoCl^{-} neurons (magenta). Scale bars: 50 µm.

i,j, Comparison of fluorescence traces (i) and grouped analysis of average calcium responses (j) in PhoCl^{+} (right) and PhoCl^{-} (left) neurons. No significant differences between two groups (n = 220 neurons). Dashed lines: median, quartiles. Statistical analysis was performed using an unpaired one-tailed Student's *t*-test.

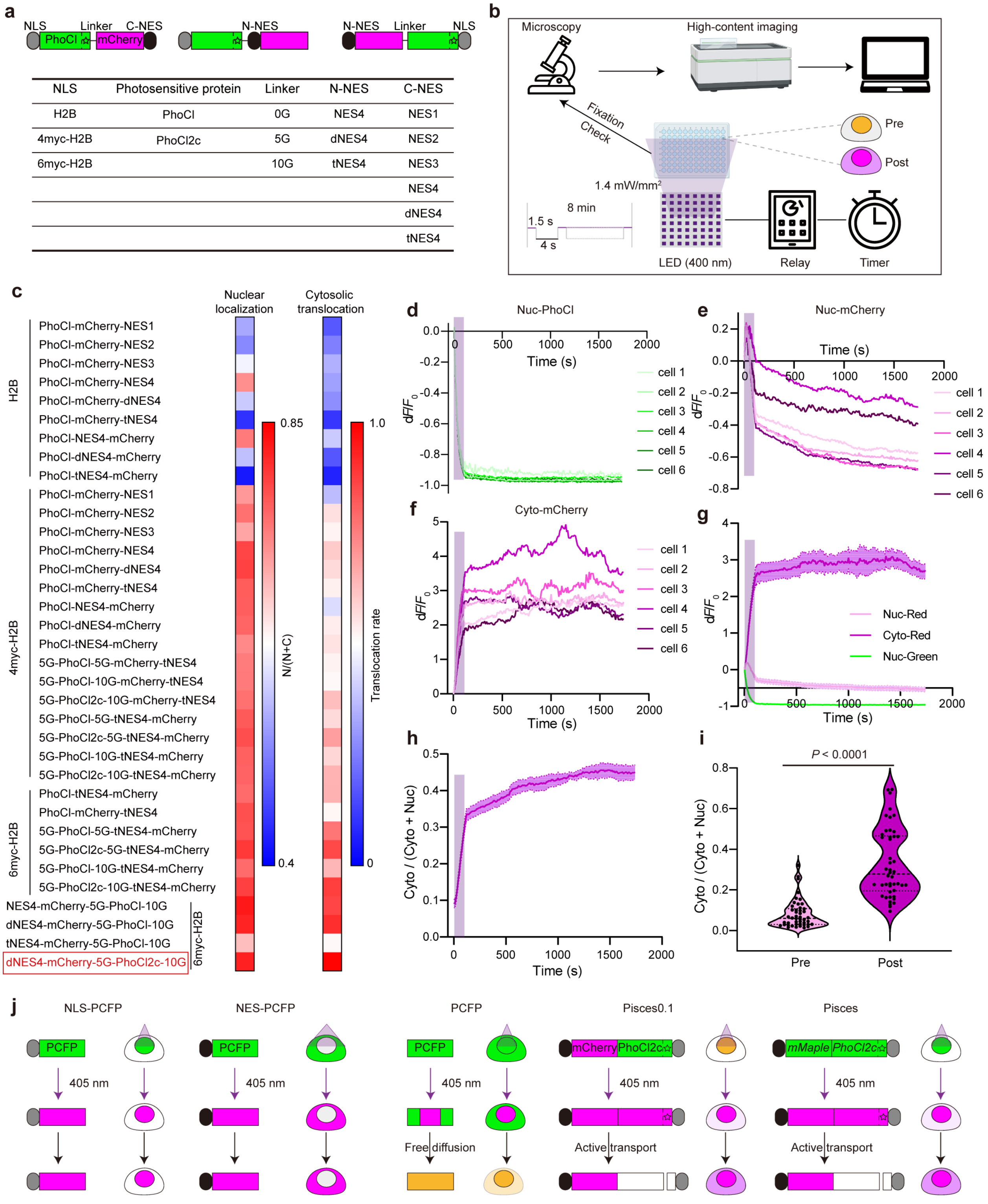

Supplementary Fig. 2. Development and *in vitro* characterization of a photo-inducible single-cell labeling system (Pisces0.1).

a, Schematic design of the light-inducible protein translocation system, which integrates PhoCl, mCherry, varying linker lengths, and various nuclear localization signal peptides (NLS) and

nuclear export signal peptides (NES). Amino acid sequences for NLS and NES are listed in the Methods.

b, Illustration of fluorescence detection in H1299 cells expressing different translocation constructs, with or without 405-nm LED activation. The stimulation pattern consisted of 1.5-s on, 4-s off for 8 min, with a power output of approximately 1.4 mW/mm².

c, Heatmap showing nuclear localization ratios and changes in translocation efficiency for 35 constructs. The nuclear localization ratio was calculated as the ratio of nuclear (N) red fluorescence to the total cell (nucleus and cytosol, N+C) red fluorescence. The translocation rate change was calculated as the difference in cytosolic localization ratios (C/(N+C)) before and after LED stimulation. Pisces0.1 (dNES4-mCherry-5G-PhoCl2c-10G-6myc-H2B) exhibits strong nuclear localization and the highest translocation efficiency, highlighted by a red rectangle (n = 3 experiments).

d-f, Normalized fluorescence traces showing changes in nuclear PhoCl (d), nuclear mCherry (e), and cytosolic mCherry (f) for six cells expressing Pisces0.1 (indicated in the Fig. 1E) with a 2-min continuous 405-nm laser scan (1.5 μW, highlighted in the purple region).

g, Average fluorescence changes in nuclear PhoCl, nuclear mCherry, and cytosolic mCherry in six cells expressing Pisces0.1.

h, Mean dynamics of cytosolic localization of mCherry in six cells expressing Pisces0.1 with the 2-min continuous 405-nm laser scan.

i, Comparison of cytosolic localization proportions of mCherry before and after 405-nm laser activation in 46 cells expressing Pisces0.1 from four independent imaging experiments. Dashed lines: median, quartiles. Statistical analysis (paired one-tailed Student's *t*-test).

j, Schematic representation illustrating the design of the constructs, activation patterns, and expected outcomes of the green-to-red photoconvertible fluorescent proteins (PCFP), including: (1) a PCFP confined to the nucleus (with an NLS, gray), (2) a PCFP excluded from the nucleus (with an NES, dark), (3) a PCFP dynamically exchanging between the cytoplasm and nucleus (with no signal peptide), (4) Pisces0.1, and (5) the final Pisces construct.

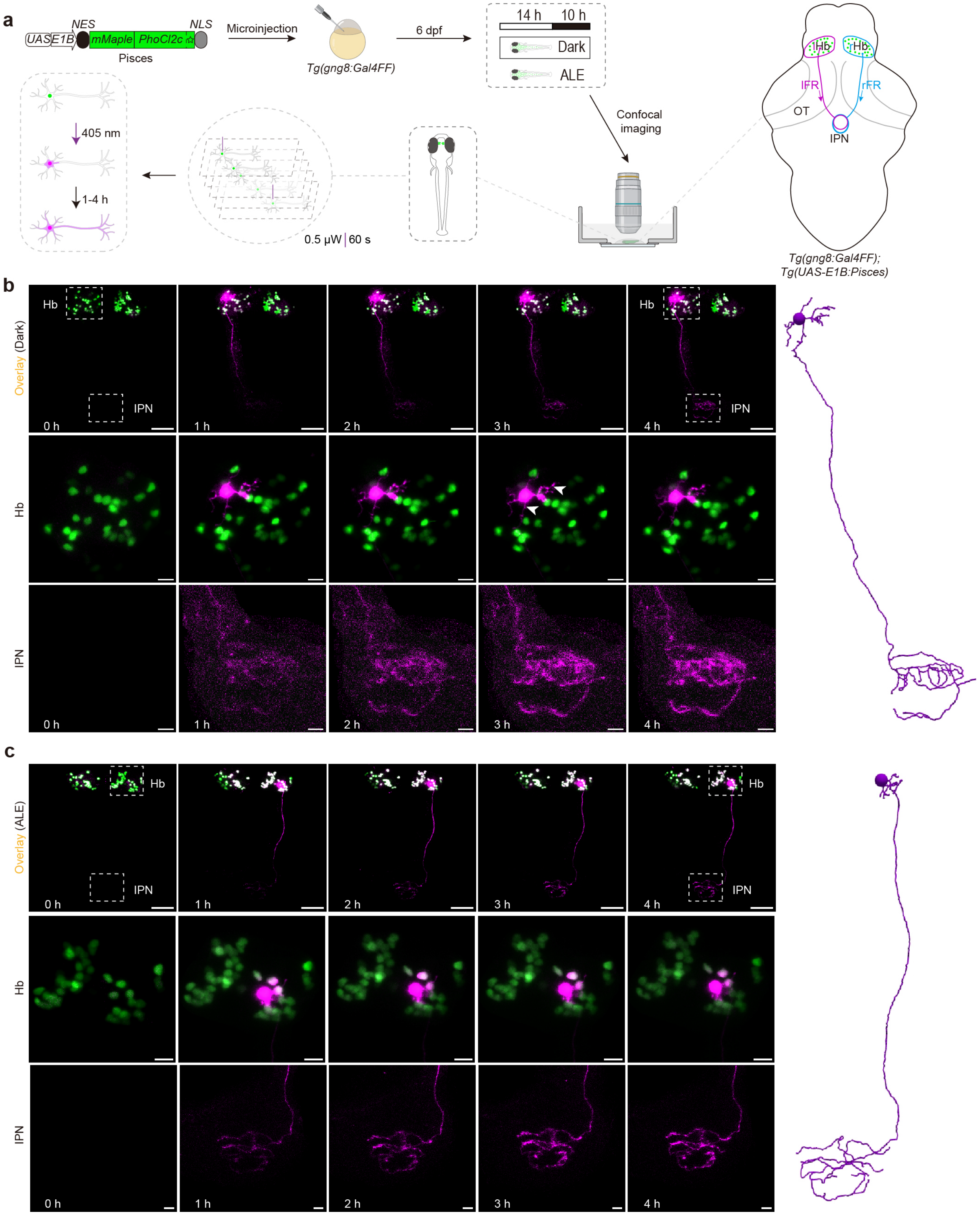

Supplementary Fig. 3. Morphological labeling of individual habenular neurons using Pisces in larval zebrafish.

a, Schematic illustrating the experimental procedure for labeling the morphology of a single habenular neuron in larval zebrafish. Nuclear Pisces was activated by a single pulse of 405-nm

laser (0.5 μW for 60 s). Larvae were raised in either constant darkness or in ALE. The typical morphological projection of a habenular neuron is shown on the right, with axonal projections extending through the fasciculus retroflexus (FR) fiber bundles to the interpeduncular nucleus (IPN).

b,c, Representative z-axis maximum projection images of habenular neurons within 4 hours in larval zebrafish raised in dark (b) and ALE (c) conditions. Full neuronal morphology was dimly labeled within 1 hour, with brightness increasing over 3 - 4 hours (upper panel). Dashed squares highlight zoomed-in views of the habenula (Hb, middle panel) and the IPN (bottom panel), were clearly visualized in both dark and ALE conditions. The brightness of the habenula was adjusted to improve the visibility of the activated neuron. Traces of the two habenular neurons at 4 hours post-activation are shown on the right. Scale bars: 50 μm for full images, 10 μm for zoomed-in views. n = 3 fish.

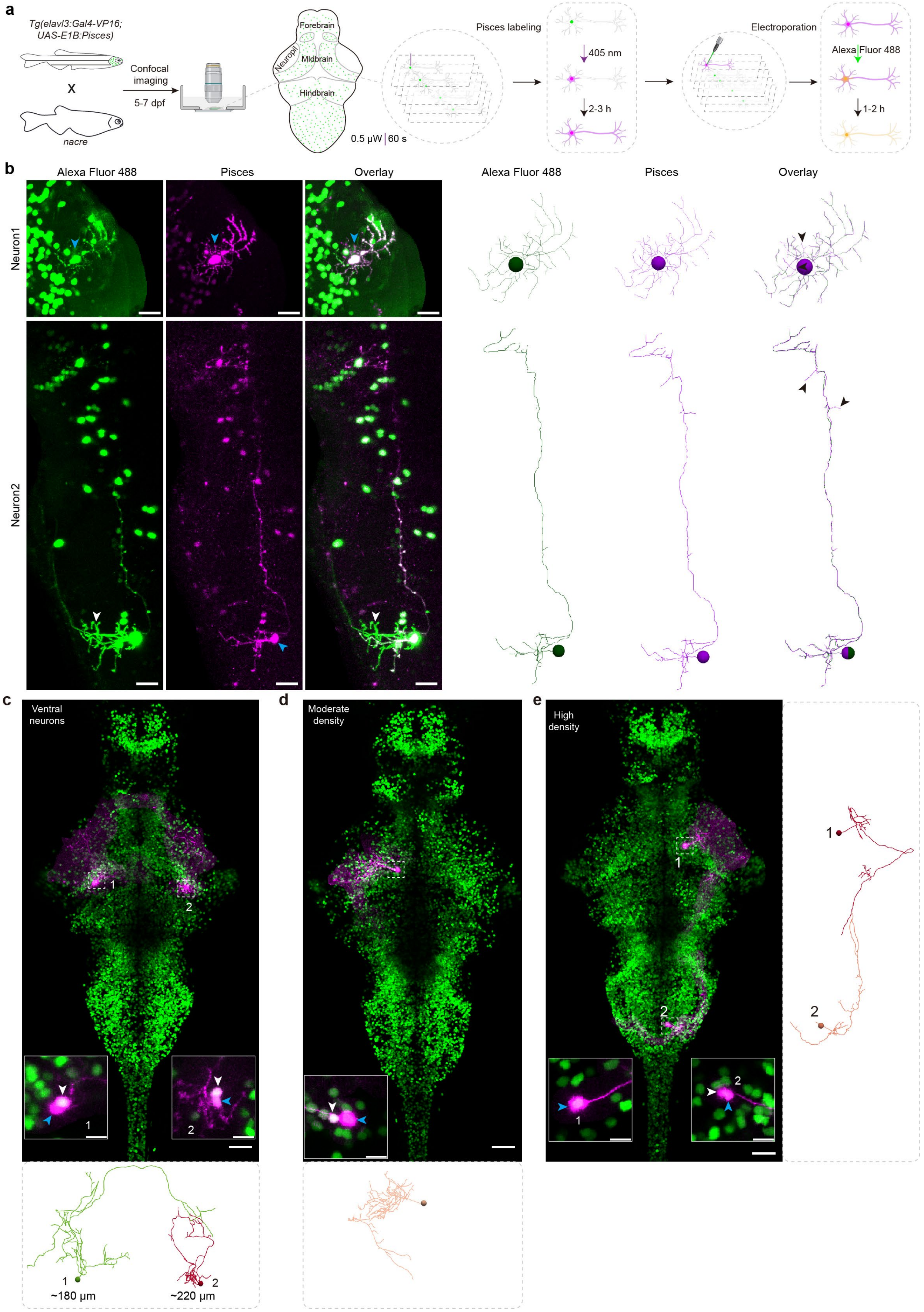
a
Tg(elavl3:Gal4-VP16;
UAS-E1B:Pisces)
X
nacre
Confocal
imaging
5-7 dpf
Forebrain
Neuropil
Midbrain
Hindbrain
0.5 μW | 60 s
Pisces labeling
405 nm
2-3 h
Electroporation
Alexa Fluor 488
1-2 h
b
Alexa Fluor 488
Pisces
Overlay
Neuron1
Neuron2
c
Ventral
neurons
1
2
~180 μm
~220 μm
d
Moderate
density
e
High
density

103

Supplementary Fig. 4. Characterization of morphological labeling performance of individual neurons in larval zebrafish.

a, Schematic illustration of the experimental procedure to label the morphology of a single neuron in larval zebrafish at 6-dpf using nuclear Pisces and single-cell electroporation. Sparse labeling by single-cell electroporation of Alexa Fluor 488 dye was performed after labeling the single neuronal morphology with Pisces under a single pulse of 405-nm laser (0.5 μW for 60 s).

b, Representative Z-axis maximum projections showing colocalization of neuronal morphology labeled by Pisces and Alexa Fluor 488. Blue arrows indicate the neuron activated for morphological tracing. White arrows (bottom) indicate a non-targeted neuron labeled during electroporation; black arrows highlight neurites missed by electroporation. Larvae were raised in constant darkness or in ambient light environment (ALE). Scale bars: 20 μm. n = 3 fish.

c, Representative Z-projection images of two neurons in ventral brain regions (depths: ~180 μm and ~220 μm), labeled as neuron 1 and 2. Dashed squares show zoomed-in views of activated neurons (bottom left/right). Blue arrows denote targeted neurons; white arrows point to neurons with mMaple activation but no PhoCl activation, resulting in red nuclei. Morphological traces are shown below. Larvae were raised under ALE conditions. Scale bars: 50 μm (full), 10 μm (zoomed). n = 3 fish.

d,e Representative Z-projections of neurons in larvae with moderate (~27%, d) and high (~40%, e) expression. In e, neurons 1 and 2 are shown, with zoomed-in views in lower corners. Blue arrows mark specifically activated neurons; white arrows indicate mMaple-positive, PhoCl-negative neurons that do not interfere with the targeted trace. Morphological reconstructions are shown below (d) and to the right (e). Larvae were raised under ALE conditions. Scale bars: 50 μm (full), 10 μm (zoomed). n = 3 fish.

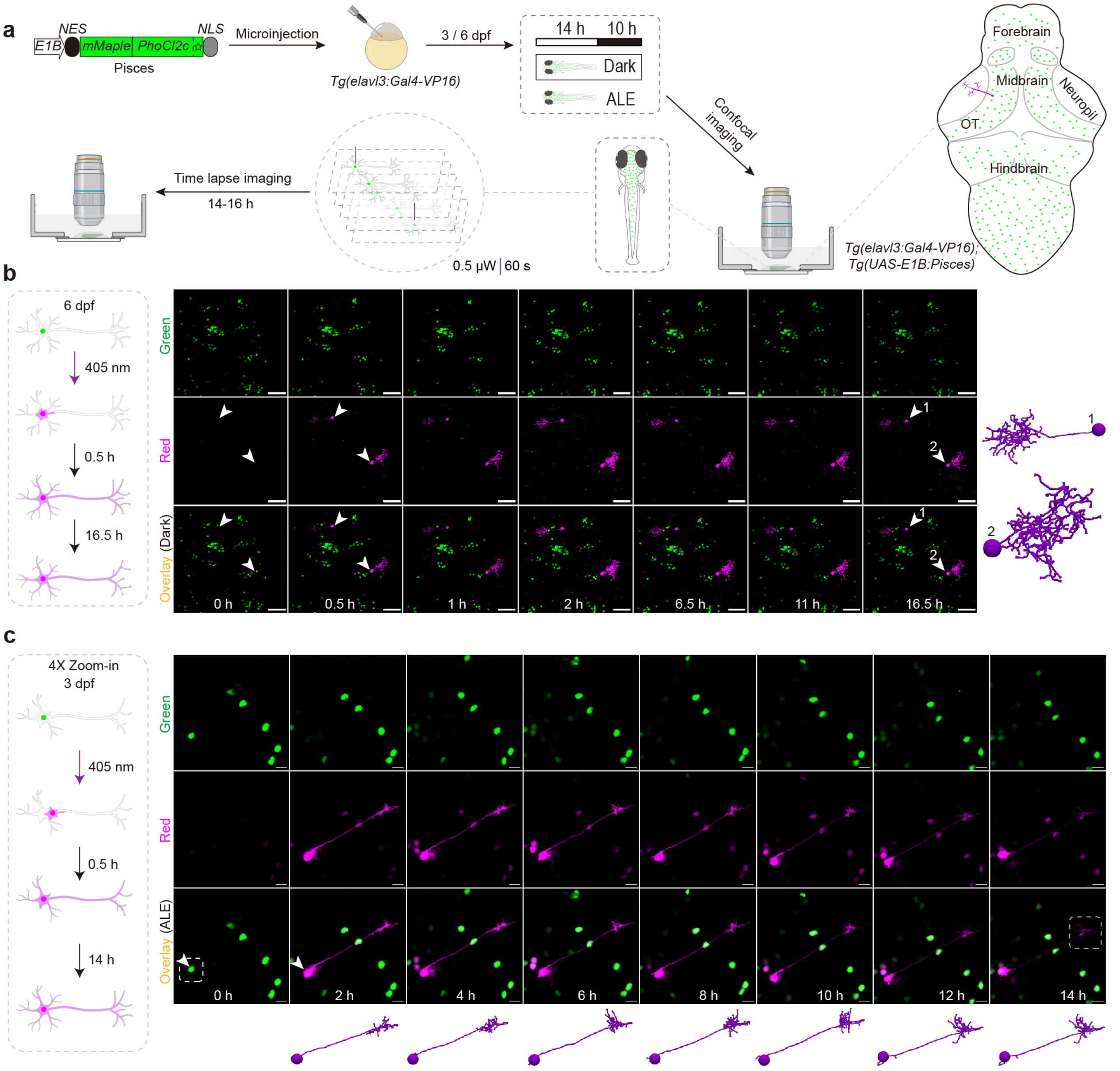

Supplementary Fig. 5. Labeling stability of Pisces in single tectal neurons in larval zebrafish. **a,** Schematic illustration of the experimental procedure to label the morphology of a single tectal neuron in larval zebrafish (3 or 6-dpf) using nuclear Pisces. A single pulse of 405-nm laser was applied (0.5 μW for 60 s). Larvae were raised either in constant darkness or in an ambient light environment (ALE). The typical morphological projection of a tectal neuron is shown on the right. **b,** Schematic illustrating the fluorescence stability of Pisces-labeled tectal neuron morphology (left) and representative z-axis maximum projection images (right) in 6-dpf zebrafish over 16 hours after a single 405-nm laser pulse (0.5 μW for 60 s). White arrowheads and numbers indicate two activated tectal neurons, showing stable fluorescence throughout the 16-hour observation. The

three-dimensional (3D) morphological traces of two neurons at 16.5 hours post-activation are shown on the right. n = 7 fish, raised in dark conditions. Scale bars: 50 µm.

c, Schematic showing the morphological dynamics of Pisces-labeled tectal neurons (left) and corresponding z-axis maximum projection images (right) over 14 hours in 3-dpf zebrafish after a 405-nm laser activation (0.5 µW for 60 s). White arrowheads indicate the activated tectal neuron, with corresponding 3D morphological traces displayed below. The dashed box highlights neurites and filopodia dynamics, shown in detail in Fig. 3c. Data are the representative from 6 fish raised in ALE conditions, showing very dim baseline fluorescence. Scale bars: 10 µm.

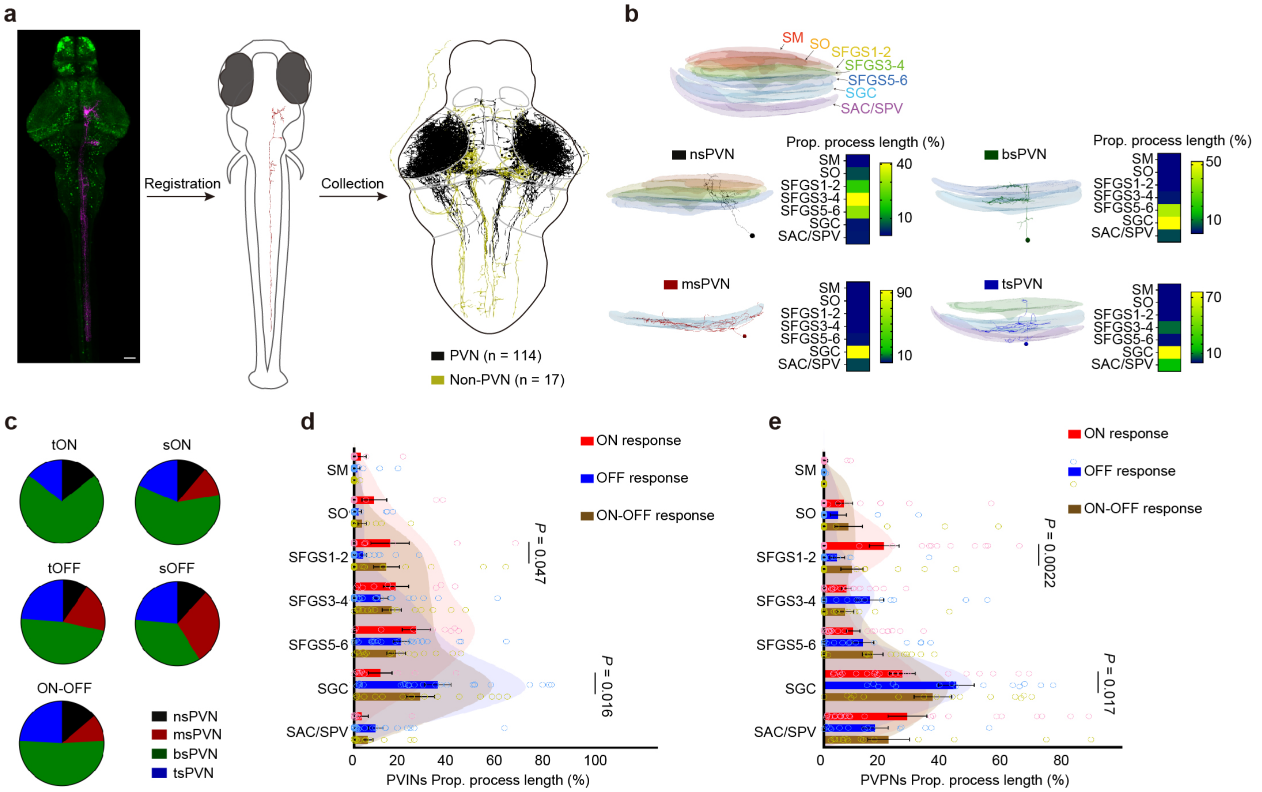

Supplementary Fig. 6. Integration of neural activities and complete morphological projections of OT neurons.

a, Schematic of the workflow for labeling neuronal morphology using Pisces, followed by tracing, reconstruction, and registration into an brain template[1]. This process identified 114 periventricular neurons (PVNs) and 17 non-PVN neurons. The registered neuronal morphologies are displayed within an outline of the zebrafish brain.

b, Neuropil layer template was reconstructed based on retinal ganglion cell projections in 6-dpf *Ki(isl2b:Gal4FF)* zebrafish larvae with sparse membrane-anchored tdTomato expression. The model comprises seven layers: SM, SO, SFGS1-2, SFGS3-4, SFGS5-6, SGC, and SAC, as indicated by arrowheads. The typical morphology and process length proportions (Prop.) of these four PVN subtypes: non-stratified (nsPVN), mono-stratified (msPVN), bi-stratified (bsPVN), and tri-stratified (tsPVN) are shown in the bottom panel. SM: stratum marginale; SO: stratum opticum; SFGS: stratum fibrosum et griseum superficiale; SGC: stratum griseum centrale; SAC: stratum album centrale; SPV: stratum periventriculare.

c, Pie chart displaying the proportions of the four PVN subtypes (nsPVN, msPVN, bsPVN, tsPVN) within five neuronal calcium response categories (tON, transient ON; sON, sustained ON; tOFF, transient OFF; sOFF, sustained OFF; ON-OFF).

d,e, Proportional (Prop.) process lengths within the neuropil region were analyzed across three types of light-responsive neurons: ON-response neurons (tON and sON), OFF-response neurons (tOFF and sOFF), and ON-OFF response neurons. These measurements were conducted separately for periventricular interneurons (PVINs, d) and periventricular projection neurons (PVPNs, e). The shaded areas are fitted and filled with polynomial curves using the polyfit function in Python. Red bar and shaded: ON response; Blue bar and shaded: OFF response; Brown bar and shaded: ON-OFF response. Two-tailed paired Student's t-test was performed to compare process length distributions across neuron response types. Error bars represent s.e.m. n = 9 (ON), 13 (OFF), 15 (ON-OFF) in d; 22 (ON), 14 (OFF), 15(ON-OFF) in e.

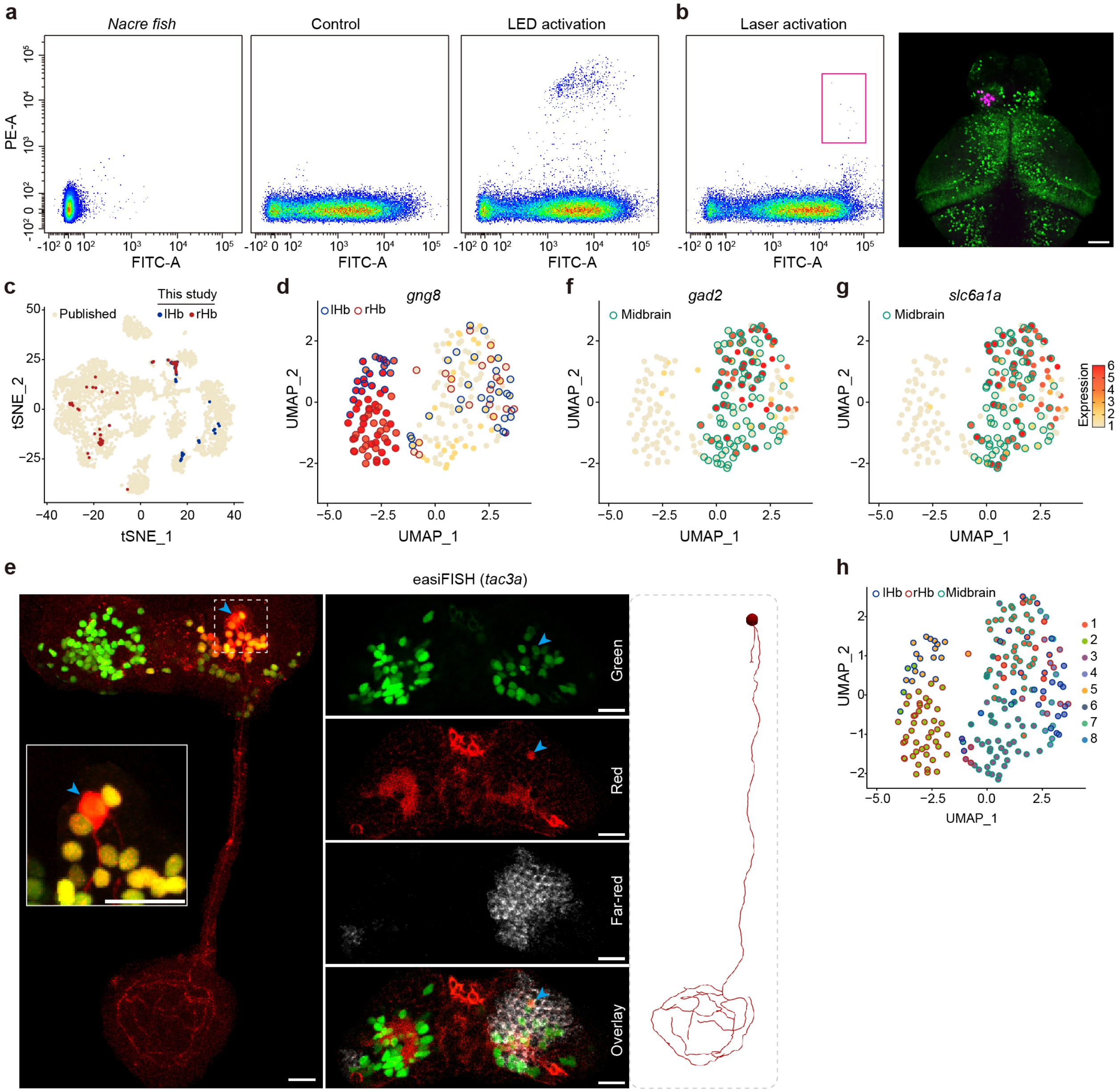

Supplementary Fig. 7. Flow cytometry and transcriptome analysis of the Pisces-activated neurons in the *Tg(elavl3:GCaMP6s)* larvae.

a, Flow cytometry analysis of brain cells from *nacre* fish (left, n = 31076) compared to neurons expressing Pisces and GCaMP6s from the *Tg(elavl3:Gal4-VP16);Tg(UAS-E1B:Pisces);Tg(elavl3:GCaMP6s)* fishline, analyzed both with (right, n = 43761) or without (medium, n = 36583) 2-min of LED illumination (1.4 mW/mm^2).

b, Flow cytometry analysis (left, n = 35192) and corresponding fluorescent images (right) of left habenular neurons activated by a single 405-nm laser pulse (0.15 μW for 10 s). A distinct

subpopulation of cells exhibiting high red fluorescence and positive green fluorescence is highlighted within a red rectangle for sorting. Scale bar: 50 μm.

c, Transcriptome data of lHb and rHb neurons from this study exhibiting strong alignment with a previously published whole habenula dataset[2].

d, Uniform manifold approximation and projection (UMAP) highlighting habenular neurons expressing the marker gene *gng8*, with lHb and rHb neurons circled in blue and red, respectively.

e, Representative z-axis maximum projection images of all slices showing *tac3a* mRNA spatial localization and single habenular neuron morphology in zebrafish larvae expressing neuronal Pisces. Left: Morphology of individual habenular neurons imaging before easiFISH. Zoomed-in view of the activated neuron (dashed square) is shown on the middle of this panel. Middle: Z-axis maximum projection images of 10 slices containing activated Pisces showing spatial expression patterns of *tac3a* mRNA detected by easiFISH, with nuclear-localized Pisces, activated neurons, and mRNA signals (Alexa Fluor 647) shown in green, red, and far-red (white), respectively. Right: Morphological reconstructions of individual neurons based on images before easiFISH. The activated neuron is indicated by blue arrows. Larvae were raised under ALE conditions. Similar results were observed from 3 larvae. Scale bars: 20 μm.

f,g, UMAP plots showing midbrain neurons expressing enriched marker genes gad2 (f) and slc6a1a (g), highlighted in green.

h, Unbiased UMAP clustering analysis of all neurons from the lHb (n = 48), rHb (n = 64) and midbrain (n = 90) identify eight distinct neuronal subpopulations.

Supplementary tables

Supplementary Table 1. Summary of the comparison of approaches for multimodal studies.

	Pisces	FuGIMA	PhOTseq	CAMPARI	Patch-Seq
Type	Non-invasive	Non-invasive	Non-invasive	Non-invasive	Invasive
Modules	Nuclear Pisces	Whole cell PAGFP	Whole cell PAmCherry	Whole cell CAMPARI	Electrode
	Cytosolic GCaMP6s	Nuclear GCaMP6	Nuclear GCaMP6		Dyes
Pattern before	Nuc green	Nuc green	Nuc green	Whole cell green	Colorless
Pattern after	Whole cell red[a]	Cell green[b]	Cell red	Whole cell green and cell red	Not mentioned
In vivo activation	One-photon (405 nm)	Two-photon (750 nm)	Two-photon (810 nm)	Two-photon (800 nm)	Not applicable
Activation pattern	Continuous	Repeated	Continuous	Continuous	Not applicable
Activation time	10 - 60 s	>30 min	>13 min	4 min	Not applicable
Laser power	Low (0.5 μW)	High (10 - 17 mW)	High (150 mW)	High (10 mW)	Not applicable
Experiment scenario - dark	Yes	Yes	Yes	Yes	Yes
Experiment scenario - ambient light environment	Yes	No	No	No	Yes
Trafficking type and rate (μm/s)	Active transport (~ 1)	Free diffusion (~ 0.02 - 0.09)	Free diffusion (~ 0.02 - 0.09)	Free diffusion (~ 0.02 - 0.09)	Free diffusion (~ 0.02 - 0.09)
Throughput	Moderate	Moderate	Moderate	Moderate	Low
Morphological labeling	Complete (> 1 mm)	Not complete (< 200 μm)	Not mentioned	Not mentioned	Not mentioned
Neuronal morphology	Any neuron Whole brain	Optical tectum, Spinal neuron	Not applicable	Not applicable	Whole brain
FACS sorting	Yes	No	Yes	Yes	Yes
Neural activity	Yes	Yes	Yes	Yes	Yes
Transcriptome	Yes	Not mentioned	Yes	Yes	Yes
Multimodal information	Three	Two	Two	Two	Three
Citation	This study	[3,4]	[5]	[6]	[7]

a: Whole cell red (green) means the complete morphology of a neuron is red (green).

b: Cell green (red) means the soma and/or major process of neuron is green (red), but it is not sure whether the morphology is entire.

Supplementary note legends.

Supplementary Note 1. The amino acids of Pisces0.1 and Pisces1.

Pisces0.1

MALQKKLEELELDEGALQKKLEELELDEGGSVSKGEEDNMAIIKEFMRFKVHMEGSVN
GHEFEIEGEGEGRPYEGTQTAKLKVTKGGPLPFAWDILSPQFMYGSKAYVKHPADIPDY
LKLSFPEGFKWERVMNFEDGGVVTVTQDSSLQDGEFIYKVKLRGTNFPSDGPVMQKKT
MGWEASSERMYPEDGALKGEIKQRLKLKDGGHYDAEVKTTYKAKKPVQLPGAYNVNI
KLDITSHNEDYTIVEQYERAEGRHSTGGMDELYKGGSGGVIPDYFKQSFPEGYSWERSM
TYEDGGICIATNDITMEGDSFINKIHFQGTNFPPNGPVMQKRTVGWEASTEKMYERDGV
LKGDVKMKLLLKGGGHYRGDYRTTYKVKQKPVKLPDCHFVDHRIEILSHDKDYNKVK
LYEHAVAKTSTDSMDELYKGGSGGMVSKGEETITSVIKPDMKNKLRMEGNVNGHAFVI
EGEGSGKPFEGIQTIDLEVKEGAPLPFAYDILTTAFHYGNRVFTKYPRGGGGGGGGGGP
AAKRVKLDPAAKRVKLDPAAKRVKLDPAAKRVKLDPAAKRVKLDPAAKRVKLDGSM
PEPAKSAPAPKKGSKKAVTKAQKKGGKKRKRSRKESYSIYVYKVLKQVHPDTGISSKA
MGIMNSFVNDIFERIAGEASRLAHYNKRSTITSREIQTAVRLLLPGELAKHAVSEGTKAIT
KYTSAK*

Pisces1

MALQKKLEELELDEGALQKKLEELELDEGGSVSKGEETIMSVIKPDMKIKLRMEGNVN
GHAFVIEGEGSGKPFEGIQTIDLEVKEGAPLPFAYDILTTAFHYGNRVFTKYPEDIPDYFK
QSFPEGYSWERSMTYEDGGICIATNDITMEEDSFINKIHFKGTNFPPNGPVMQKRTVGWE
VSTEKMYVRDGVLKGDVKMKLLLKGGSHYRCDFRTTYKVKQKAVKLPDYHFVDHRIE
ILSHDKDYNKVKLYEHAVARNSTDSMDELYKGGSGGVIPDYFKQSFPEGYSWERSMTY
EDGGICIATNDITMEGDSFINKIHFQGTNFPPNGPVMQKRTVGWEASTEKMYERDGVLK
GDVKMKLLLKGGGHYRGDYRTTYKVKQKPVKLPDCHFVDHRIEILSHDKDYNKVKLY
EHAVAKTSTDSMDELYKGGSGGMVSKGEETITSVIKPDMKNKLRMEGNVNGHAFVIEG
EGSGKPFEGIQTIDLEVKEGAPLPFAYDILTTAFHYGNRVFTKYPRGGGGGGGGGGPAA
KRVKLDPAAKRVKLDPAAKRVKLDPAAKRVKLDPAAKRVKLDPAAKRVKLDGSMPEP
AKSAPAPKKGSKKAVTKAQKKGGKKRKRSRKESYSIYVYKVLKQVHPDTGISSKAMGI
MNSFVNDIFERIAGEASRLAHYNKRSTITSREIQTAVRLLLPGELAKHAVSEGTKAITKYT
SAK*

Supplementary References

1. Du, X.-F. *et al.* Central nervous system atlas of larval zebrafish constructed using the morphology of single excitatory and inhibitory neurons. *bioRxiv* 2025.06.06.658008 (2025) doi:10.1101/2025.06.06.658008.
2. Pandey, S., Shekhar, K., Regev, A. & Schier, A. F. Comprehensive Identification and Spatial Mapping of Habenular Neuronal Types Using Single-cell RNA-seq. *Curr. Biol.* **28**, 1052-1065.e7 (2018).
3. Kramer, A., Wu, Y., Baier, H. & Kubo, F. Neuronal Architecture of a Visual Center that Processes Optic Flow. *Neuron* **103**, 118-132.e7 (2019).
4. Förster, D., Kramer, A., Baier, H. & Kubo, F. Optogenetic precision toolkit to reveal form, function and connectivity of single neurons. *Methods* **150**, 42–48 (2018).
5. Lee, D., Kume, M. & Holy, T. E. Sensory coding mechanisms revealed by optical tagging of physiologically defined neuronal types. *Science* **366**, 1384–1389 (2019).
6. Fosque, B. F. *et al.* Labeling of active neural circuits in vivo with designed calcium integrators. *Science* **347**, 755–760 (2015).
7. Cadwell, C. R. *et al.* Electrophysiological, transcriptomic and morphologic profiling of single neurons using Patch-seq. *Nat. Biotechnol.* **34**, 199–203 (2016).
