# Supplementary material for: Designed optogenetic tool for bridging single-neuronal multimodal information in intact animals
Source: Nat Commun. 2025 Aug 20;16:7764. doi: 10.1038/s41467-025-62938-w (PMC12368189; doi:10.1038/s41467-025-62938-w)
Supplement: Supplementary file 2 — Description of Additional Supplementary Information [file 41467_2025_62938_MOESM2_ESM.pdf]

Description of Additional Supplementary Files

File Name: Supplementary Movie 1
Description: **3D reconstruction of a 6-dpf larval zebrafish expressing Pisces0.1 and Pisces, captured before and after 2 min of LED stimulation. Relative to Fig. 2d,e.** High-resolution confocal imaging was conducted on approximately 40 central optical sections of larval zebrafish, primarily capturing dorsal and mid-regional neurons. While ventral neurons were also activated and traced by Pisces during the experiment, they were not included in the imaging field due to the protocol.

File Name: Supplementary Movie 2
Description: **3D reconstruction of habenular neuron projections activated bilaterally in a 6-dpf zebrafish raised in the dark. Relative to Fig. 3c.** Note that the two fluorescent somata observed in each hemisphere in this Movie do not represent dual-neuron labeling (Fig. 3c). Specifically, these signals arise from: (1) a fully labeled habenular neuron, and (2) an adjacent neuron with partial nuclear-localized mMaple activation (Fig. 3c). This optical configuration (3D reconstruction at identical contrast settings) simultaneously shows both neuronal somata and their extending neurites, which may create the illusion of dual labeling.

File Name: Supplementary Movie 3
Description: **3D reconstruction of singleneuron labeling through Pisces and single-cell electroporation in 6-dpf larvae raised in the ALE. Relative to Supplementary Fig. 4b.** The Movie shows colocalization of the same neuronal morphology labeled by single-cell electroporation using Alexa Fluor 488 and by Pisces, highlighting the completeness of Pisces labeling. One additional neuron labeled during single-cell electroporation of Alexa Fluor 488 is visible in the right panel.

File Name: Supplementary Movie 4
Description: **3D reconstruction of a locus coeruleus norepinephrine (LC-NE) neuron projection (bottom) in a 6-dpf zebrafish raised in the dark. Relative to Fig. 3d.** The image was taken 4 hours post-activation using a 1-min stimulation of 405-nm laser (0.5 µW). In most regions, the overlays show strong correspondence between the Pisces signals and the skeletons of the traced projections. However, we note that in some areas, particularly where signal intensity is low, the fluorescent signal may appear faint or unclear when contrasted with the full-frame image.

File Name: Supplementary Movie 5
Description: **3D reconstruction of the morphology of two ventral neurons labeling with Pisces in a 6-dpf zebrafish raised in the ALE. Relative to Supplementary Fig. 4c.** This Movie presents fluorescent images of two ventral neurons, which are ~180 µm and ~220 µm depth from the dorsal slice, respectively. Neuron activation was performed using a 405-nm laser (0.5 µW for 10 seconds, applied multiple times).

File Name: Supplementary Movie 6
Description: **3D reconstruction of neurons activated in Tg(elavl3:Gal4-VP16;UAS-E1B:Pisces) larvae with moderate or dense expressing population. Relative to**

Supplementary Fig. 4d,e. The Movie shows the morphology of activated neurons in zebrafish larvae with moderate (~27%) and high (~40%) Pisces expression, with larvae raised under ambient light conditions. Neuron activation was precisely performed by applying a 405-nm laser at 0.5 µW for 10 seconds, with multiple repetitions.

File Name: Supplementary Movie 7
Description: **Time-lapse imaging of a tectal neuron in a 3-dpf larval zebrafish expressing Pisces, showing morphological changes over 14 hours. Relative to Fig. 3e.** Neuron activation was conducted by a 405-nm laser (0.5 µW for 60 s), with the fish raised in an ambient light environment.

File Name: Supplementary Movie 8
Description: **3D reconstruction showing two adjacent tectal neurons sequentially activated in a 6-dpf zebrafish, raised in ambient light conditions. Relative to Fig. 4b,c.** Images were captured 4 hours post 405-nm laser activation at 0.5 µW for 60 s.

File Name: Supplementary Movie 9
Description: **3D reconstruction of the morphological projections of 9 individual neurons across the brain and spinal cord of a 6-dpf larval zebrafish raised in the dark. Relative to Fig. 4d,e.** Each neuron was activated with a total time of 30 - 60 s of 405-nm laser (0.5 µW), with imaging captured 4 hours postactivation.

File Name: Supplementary Movie 10
Description: **3D reconstruction of three periventricular interneurons (PVINs) displaying distinct calcium response patterns to red light stimulation: transient ON (top), sustained ON (medium), and transient OFF (bottom). Relative to Fig. 5b.** Calcium activity was monitored using GCaMP6s, followed by Pisces activation with a 405-nm laser at 0.5 µW for 60 s. Imaging was conducted at 4 hours post-activation.

File Name: Supplementary Movie 11
Description: **3D reconstruction of two periventricular projection neurons (PVPNs) exhibiting sustained OFF (top) and ON-OFF (bottom) calcium responses to red light. Relative to Fig. 5b.** Calcium activity was monitored using GCaMP6s, followed by Pisces activation with a 405-nm laser at 0.5 µW for 60 s. Imaging was conducted at 4 hours post-activation.

File Name: Supplementary Movie 12
Description: **3D reconstruction of the expression pattern of gng8 mRNA in a Tg(elavl3:Gal4- VP16;UAS-E1B:Pisces) larva at 6-dpf. Relative to Fig. 6g.** The left panel shows the morphology of individual habenular neuron with projections into the IPN, activated using a 405-nm laser at 0.5 µW for 60 seconds. The right panel shows the spatial expression patterns of gng8 mRNA detected using easiFISH. The larva was raised under ALE conditions.

File Name: Supplementary Movie 13
Description: **3D reconstruction of the expression pattern of tac3a mRNA in a Tg(elavl3:Gal4- VP16;UAS-E1B:Pisces) larva at 6-dpf. Relative to Supplementary Fig. 7h.**

The left panel of the Movie shows the morphology of a right habenular neuron, while the right panel displays the spatial expression patterns of tac3a mRNA (Alexa Fluor 647) along with nuclear-localized Pisces and activated neurons. The larva was raised under ALE conditions.
