# Supplementary material for: Designed optogenetic tool for bridging single-neuronal multimodal information in intact animals
Source: Nat Commun. 2025 Aug 20;16:7764. doi: 10.1038/s41467-025-62938-w (PMC12368189; doi:10.1038/s41467-025-62938-w)
Supplement: Supplementary file 16 — Reporting Summary [file 41467_2025_62938_MOESM16_ESM.pdf]

nature portfolio

Corresponding author(s): Rong-kun Tao, Jiulin-Du

Last updated by author(s): Jul 24, 2025

Reporting Summary

Nature Portfolio wishes to improve the reproducibility of the work that we publish. This form provides structure for consistency and transparency in reporting. For further information on Nature Portfolio policies, see our Editorial Policies and the Editorial Policy Checklist.

Statistics

For all statistical analyses, confirm that the following items are present in the figure legend, table legend, main text, or Methods section.

n/a	Confirmed	
☐	☒	The exact sample size (*n*) for each experimental group/condition, given as a discrete number and unit of measurement
☐	☒	A statement on whether measurements were taken from distinct samples or whether the same sample was measured repeatedly
☐	☒	The statistical test(s) used AND whether they are one- or two-sided<br>*Only common tests should be described solely by name; describe more complex techniques in the Methods section.*
☒	☐	A description of all covariates tested
☒	☐	A description of any assumptions or corrections, such as tests of normality and adjustment for multiple comparisons
☐	☒	A full description of the statistical parameters including central tendency (e.g. means) or other basic estimates (e.g. regression coefficient) AND variation (e.g. standard deviation) or associated estimates of uncertainty (e.g. confidence intervals)
☐	☒	For null hypothesis testing, the test statistic (e.g. *F*, *t*, *r*) with confidence intervals, effect sizes, degrees of freedom and *P* value noted<br>*Give P values as exact values whenever suitable.*
☒	☐	For Bayesian analysis, information on the choice of priors and Markov chain Monte Carlo settings
☒	☐	For hierarchical and complex designs, identification of the appropriate level for tests and full reporting of outcomes
☒	☐	Estimates of effect sizes (e.g. Cohen's *d*, Pearson's *r*), indicating how they were calculated

Our web collection on statistics for biologists contains articles on many of the points above.

Software and code

Policy information about availability of computer code

Data collection	Harmony high content imaging and analysis software of Operetta CLS (PerkinElmer)<br>The FLUOVIEW FV31S-SW software of FV3000 automatic confocal laser scanning microscope (Olympus)
Data analysis	Graphpad Prim (V9.5.1), MATLAB R2018a (Mathworks), ImageJ 1.53c (NIH), previously published code for fluorescent image analysis (https://github.com/KeJiiii/FIQA/releases), Imaris X64 9.0.1 for reconstruction of raw neuronal morphology images in 3-dimensional (3D) view. The custom 3D visualization software used in this study was developed using the ParaView framework and is currently under active development in our laboratory. For collaborative or academic use prior to publication, please contact the lead corresponding author directly with a detailed request.

For manuscripts utilizing custom algorithms or software that are central to the research but not yet described in published literature, software must be made available to editors and reviewers. We strongly encourage code deposition in a community repository (e.g. GitHub). See the Nature Portfolio guidelines for submitting code & software for further information.

Data

Policy information about availability of data

All manuscripts must include a data availability statement. This statement should provide the following information, where applicable:

- Accession codes, unique identifiers, or web links for publicly available datasets
- A description of any restrictions on data availability
- For clinical datasets or third party data, please ensure that the statement adheres to our policy

The single cell transcriptome datasets have been deposited to the National Genomics Data Center (accession number: PRJCA031958). The whole habenula dataset can be accessed on the Gene Expression Omnibus (GEO) under accession code GSE105115 (https://www.ncbi.nlm.nih.gov/geo/query/acc.cgi?acc=GSE105115). All data displayed is available upon request. Source data are provided with this paper. Zebrafish lines and relevant plasmids generated in this study are available upon request. The plasmid pminiTol2-UAS-E1B-Pisces is also available on the WeKwikGene plasmid repository at Westlake Laboratory, China (https://wekwikgene.wllsb.edu.cn/).

Research involving human participants, their data, or biological material

Policy information about studies with human participants or human data. See also policy information about sex, gender (identity/presentation), and sexual orientation and race, ethnicity and racism.

Reporting on sex and gender	N/A
Reporting on race, ethnicity, or other socially relevant groupings	N/A
Population characteristics	N/A
Recruitment	N/A
Ethics oversight	N/A

Note that full information on the approval of the study protocol must also be provided in the manuscript.

Field-specific reporting

Please select the one below that is the best fit for your research. If you are not sure, read the appropriate sections before making your selection.

☒ Life sciences ☐ Behavioural & social sciences ☐ Ecological, evolutionary & environmental sciences

For a reference copy of the document with all sections, see nature.com/documents/nr-reporting-summary-flat.pdf

Life sciences study design

All studies must disclose on these points even when the disclosure is negative.

Sample size	No statistical methods were used to predetermine the sample size. The sample size (n) of each experiment is provided in the figure legends.
Data exclusions	No data were excluded from the analysis.
Replication	The replication number is indicated in the legend of corresponding figures or source data where applicable. For all the data, at least three independent experiments were carried out with similar results.
Randomization	Cells and animals were randomly assigned into control or experimental groups.
Blinding	The investigators were not blinded to group allocation during data collection and analysis. The analysis was performed objectively and not subjective to human bias.

Reporting for specific materials, systems and methods

We require information from authors about some types of materials, experimental systems and methods used in many studies. Here, indicate whether each material, system or method listed is relevant to your study. If you are not sure if a list item applies to your research, read the appropriate section before selecting a response.

Materials & experimental systems

n/a	Involved in the study
☒	☐ Antibodies
☐	☒ Eukaryotic cell lines
☒	☐ Palaeontology and archaeology
☐	☒ Animals and other organisms
☒	☐ Clinical data
☒	☐ Dual use research of concern
☒	☐ Plants

Methods

n/a	Involved in the study
☒	☐ ChIP-seq
☐	☒ Flow cytometry
☒	☐ MRI-based neuroimaging

Eukaryotic cell lines

Policy information about cell lines and Sex and Gender in Research

Cell line source(s)	H1299 cells (TCHu160) and HEK293 cells (GNHu43) were from the cell bank of Chinese Academy of Sciences.
Authentication	Cells were validated based on the morphology under microscope.
Mycoplasma contamination	Cells were routinely tested for mycoplasma.
Commonly misidentified lines (See ICLAC register)	This study did not involve commonly misidentified lines.

Animals and other research organisms

Policy information about studies involving animals; ARRIVE guidelines recommended for reporting animal research, and Sex and Gender in Research

Laboratory animals	Danio rerio, Nacre and AB strain. Studies were conducted on larval zebrafish from 3-7 days post-fertilization (dpf).
Wild animals	No wild animals were used in the study.
Reporting on sex	Sex is not defined in 3-7 dpf zebrafish, therefore there are no delineations of sex.
Field-collected samples	No field collected samples were used in the study.
Ethics oversight	All procedures involving animals were done in compliance with the Animal Care of Chinese Academy of Sciences and Use Committee and approved by Chinese Academy of Sciences (Approval no. NA-046–2019).

Note that full information on the approval of the study protocol must also be provided in the manuscript.

Plants

Seed stocks	N/A
Novel plant genotypes	N/A
Authentication	N/A

Flow Cytometry

Plots

Confirm that:

☒ The axis labels state the marker and fluorochrome used (e.g. CD4-FITC).

☒ The axis scales are clearly visible. Include numbers along axes only for bottom left plot of group (a 'group' is an analysis of identical markers).

☒ All plots are contour plots with outliers or pseudocolor plots.

☒ A numerical value for number of cells or percentage (with statistics) is provided.

Methodology

Sample preparation	We used zebrafish at 6-dpf for FACS experiments. Neurons were selectively activated in one of three brain regions: the left habenula, right habenula, or midbrain. Each larva had neurons activated in only one brain region. Activated larvae were anesthetized using 0.2% MS-222 (Sigma) and placed on a soft agar plate immersed in pre-cooled freshly prepared choline chloride solution. The dorsal skull and eyes were carefully removed with forceps, followed by incisions were made caudal to the olfactory bulbs and rostral to the midbrain to isolate the brain tissue. The dissected brains were transferred into choline chloride solution containing 20 U/mL papain and 100 U/mL DNase I (Worthington) at 37°C for 20 min, followed by gentle trituration with fire-polished glass Pasteur pipettes to create a single-cell suspension. To remove any remaining cell aggregates, the suspension was passed through a 40 µm cell strainer (Nunc).
Instrument	BD FACS Aria Fusion Flow Cytometer (BD Biosciences)
Software	FlowJo V10
Cell population abundance	Zebrafish brain were dissected and brain cells were used at desired proportions for sorting experiments.
Gating strategy	Debris and dead cells were removed with an initial FSC/SSC gate.

☒ Tick this box to confirm that a figure exemplifying the gating strategy is provided in the Supplementary Information.
